# Exploring Health Trends Prior to State Pension Age for The Netherlands up to 2040

**DOI:** 10.3390/ijerph19074209

**Published:** 2022-04-01

**Authors:** Maaike van der Noordt, Johan J. Polder, Marjanne H. D. Plasmans, Henk B. M. Hilderink, Dorly J. H. Deeg, Theo G. van Tilburg, Suzan van der Pas, Fons van der Lucht

**Affiliations:** 1 Department of Health Knowledge Integration, Center for Health and Society, National Institute for Public Health and the Environment (RIVM), 3720 BA Bilthoven, The Netherlands; j.j.polder@tilburguniversity.edu (J.J.P.); marjanne.plasmans@rivm.nl (M.H.D.P.); henk.hilderink@rivm.nl (H.B.M.H.); fons.van.der.lucht@rivm.nl (F.v.d.L.); 2 Department of Epidemiology and Biostatistics, Amsterdam Public Health Research Institute, Amsterdam UMC, Vrije Universiteit Amsterdam, 1081 HV Amsterdam, The Netherlands; djh.deeg@amsterdamumc.nl (D.J.H.D.); pas.vd.s@hsleiden.nl (S.v.d.P.); 3 Tilburg School of Social and Behavioral Sciences, Tilburg University, Tranzo, 5000 LE Tilburg, The Netherlands; 4 Department of Sociology, Faculty of Social Sciences, Vrije Universiteit, 1081 HV Amsterdam, The Netherlands; theo.van.tilburg@vu.nl; 5 Faculty of Social Work and Applied Psychology, University of Applied Sciences Leiden, 2333 CK Leiden, The Netherlands; 6 Department of Public Health and Primary Care, Leiden University Medical Centre, 2333 ZD Leiden, The Netherlands; 7 Centre of Expertise Healthy Ageing, Hanze University of Applied Sciences, 9747 AA Groningen, The Netherlands

**Keywords:** extension of working lives, self-rated health, functional limitations, forecasting health

## Abstract

Background: In many Western countries, the state pension age is being raised to stimulate the extension of working lives. It is not yet well understood whether the health of older adults supports this increase. In this study, future health of Dutch adults aged 60 to 68 (i.e., the expected state pension age) is explored up to 2040. Methods: Data are from the Dutch Health Interview Survey 1990–2017 (N ≈ 10,000 yearly) and the Dutch Public Health Monitor 2016 (N = 205,151). Health is operationalized using combined scores of self-reported health and limitations in mobility, hearing or seeing. Categories are: good, moderate and poor health. Based on historical health trends, two scenarios are explored: a stable health trend (neither improving nor declining) and an improving health trend. Results: In 2040, the health distribution among men aged 60–68 is estimated to be 63–71% in good, 17–28% in moderate and 9–12% in poor health. Among women, this is estimated to be 64–69%, 17–24% and 12–14%, respectively. Conclusions: This study’s explorations suggest that a substantial share of people will be in moderate or poor health and, thus, may have difficulty continuing working. Policy aiming at sustainable employability will, therefore, remain important, even in the case of the most favorable scenario.

## 1. Background

In many Western countries, the state pension age is being raised to stimulate the extension of working lives and to contribute to financial sustainability of state pensions [1]. In the Netherlands, the state pension age is linked to the life expectancy at age 65 years (LE65) with a factor of two thirds from 2023 onwards. This means that with each year that LE65 increases, the state pension age will increase by eight months. In 2016, the state pension age was 65 and six months. By 2040, the state pension age is expected to be 68 years. It is not yet known whether the health status of the 60+ population supports this increase. In this study, the health of Dutch adults aged 60 to 68 is explored from 2016 up to 2040.

European studies projecting future health show that the prevalence of (chronic) diseases, such as arthrosis, diabetes, COPD, cancer and obesity is likely to increase in the coming years [2,3,4,5]. However, subjective health outcomes are expected to improve. The Dutch Public Health Foresight Study shows that among adults aged ≥45, an increasing proportion is expected to experience their health as good and is expected to report no functional limitations [5,6]. In line with the Dutch Public Health Foresight Study, this study focusses on the subjective health outcomes self-reported health and functional limitations because these health outcomes are more strongly associated to labor participation compared to having a chronic disease [7].

So far, future health has not been explored specifically for the older working-age population (age 60 to the state pension age). Based on historical health trends, which form the basis for the exploration of future health, there is reason to believe that the future health trend of this age category will be less favorable. Six studies in different countries show that the historical health trends of the 60–65-year-olds do not follow the same, more positive, trends of the 65+ [8,9,10,11,12,13]. Only one German study showed that there was an improvement in physical functioning among 50–65-year-olds from 1997 to 2011 [14]. Findings from a European study from 2004–2013 show that disability trends among 50–64-year-olds differ across indicators and countries, with the Dutch subsample showing a stable (unchanged) trend according to all indicators [15]. Another Dutch study shows that the number of physical limitations of 55–65-year-olds increased between 1992 and 2002, but remained stable between 2002 and 2012 [16].

It is not well understood why health trends of 60–65-year-olds are not as positive as of the 65+. Steiber et al. suggest that increases in employment rates and extension of working lives may have contributed to the stable trend [12]. With regard to the Dutch situation, it is plausible that the extension of working lives explains why there was no improvement in health among 50–64-year-olds. From 2006 to 2017, employment rates of 60–65-year-olds have increased with 31 percentage points and the actual retirement age increased from age 61.0 to age 64.6 [17,18]. Working until higher ages may have negative health effects, as older workers need more time to recover from performing physically or psychosocially demanding tasks. Prolonged exposure to these tasks may lead to health problems [19,20]. In addition, the extension of working lives is accompanied by an increase of the proportion of workers who also provide informal care [21]. Combining these tasks may cause a double burden for older workers, which affects their health status [22].

For future health trends of the older working-age population, we suggest two likely scenarios. On the one hand, it can be expected that the stable health trend will continue and that this will also be realistic for the 65–68-year-olds. The lack of improvement would be due to the extension of working lives as a result of the expected increase of the average retirement age, and the expected increase of the demand for informal care in this age category [23,24,25]. On the other hand, it can be expected that the health of 60–65-year-olds will improve, just as is expected for the surrounding ages according to the Dutch Public Health Foresight Study [6]. This might occur if adverse health effects of extended working lives do not exist or have been temporary. Several measures to encourage the extension of working lives have followed shortly after each other in the early 2010s, and the retirement age that older workers had planned on has repeatedly and unexpectedly been delayed. As a result, older workers were confronted with extensions of their working lives for which they were unable to prepare themselves. This situation may have had adverse health effects [26]. Currently, there is a well-defined plan of raising the Dutch state pension age in the coming years, for which older workers can prepare long enough beforehand [27].

The aim of this study is to explore the health of Dutch adults aged 60 to 68 years up to 2040. We assume that the two scenarios of a stable health trend (i.e., neither improving nor declining) and an improving health trend indicate a bandwidth between which health will develop. To estimate the scenarios, projections of the proportion of men and women aged 60–68 in good, moderate and poor health are made until 2040. Subsequently, the projections are expressed in the absolute number of persons over 60 years of age up until the state pension age in each health category for 2030 and 2040.

## 2. Material and Methods

### 2.1. Study Samples

Data are used from the Dutch Public Health Monitor (PHM) 2016 and the Dutch Health Interview Survey (HIS) 1990–2017 [28,29]. The PHM is a repeated cross-sectional health survey of the Community Health Services, Statistics Netherlands and the National Institute for Public Health and the Environment, conducted in 2012 and 2016. In 2016, a random sample of non-institutionalized individuals aged ≥19 years was invited (N ≈ 460,000). For the analyses, respondents aged 55–75 were included who provided information on health and limitations (N = 205,151). There were 4474 missing self-reports regarding health and limitations (2%).

The HIS is an annual cross-sectional health survey conducted by Statistics Netherlands since 1981. The sample consists of approximately 10,000 non-institutionalized persons (all ages) each year (in the years 2010–2013, approximately 15,000 persons). For the analyses, respondents aged ≥16 years were included who participated between 1990 and 2017, responded to both parts of the questionnaire, and provided information on health and limitations (N = 172,599). There were 48,128 missing self-reports regarding health and limitations (22%). Approximately 20% was missing because the participants did not respond to the second part of the questionnaire in which functional limitations was included, and approximately 2% did not provide information on their health status for unknown reasons.

### 2.2. Variables

#### 2.2.1. Demographics

Age in five-year categories based on birth year (PHM) or interview date (HIS) and sex are used as demographic variables.

#### 2.2.2. Health

Health is operationalized using a combination of two indicators: self-reported health and limitations in mobility, hearing or seeing. Self-reported health was assessed with one question: ‘How is your health in general?’. Responses ‘very good’ and ‘good’ are defined as ‘healthy’ (versus ‘fair’, ‘poor’ or ‘very poor’) [30]. Functional limitations were assessed using seven items of the OECD-indicator: Able to: carry an object of five kilograms; pick up something from the floor from an upright position; walk 400 m without stopping; follow a conversation in a group of three or more persons; have a conversation with one person; read the small print in the newspaper; recognize somebody’s face at a distance of four meters. Response options are: ‘with no difficulty’, ‘with some difficulty’, ‘with a lot of difficulty’, ‘not at all’. The indicator scores ‘healthy’ if the answers to all seven questions are ‘with no difficulty’ or ‘with some difficulty’ [31]. The combined health indicator for this study consists of three categories: good health (healthy according to both indicators), moderate health (healthy according to only one indicator) and poor health (unhealthy according to both indicators).

Both indicators, self-reported health and limitations in mobility, hearing or seeing are related to reduced productivity and early work exit [7,32]. The combined health indicator was chosen because in a preliminary analysis in PHM, it proved to be more strongly associated to labor market participation (expressed in currently working, non-working, receiving disability pension and retired) compared to one single indicator: the variance explained (Nagelkerke R2) was 18% for the combined health indicator, compared to 15% and 11% for self-reported health and limitations, respectively. At each step in the tripartite classification (from good health to moderate health, and from moderate health to poor health), the probability of labor force participation decreases.

## 3. Calculation

### 3.1. Main Analyses

Scenario A, a stable trend, is implemented as follows. First, health of 55–75-year-old men and women is assessed in five-year age categories using the PHM 2016. In 2016, the state pension age was 66.5; in 2040, it will be 68.0. Second, average health of 60–65-year-olds of PHM 2016 was applied to 60–68-year-olds in 2040. As it was observed that the 65–70-year-olds were healthier compared to the 60–65-year-olds, possibly attributable to adverse effects of the extension of working lives, the health of the 65–68-year-olds was thus also considered to be affected by these adverse effects. Third, to calculate health status for ages 60 up to the state pension age (Table 1) for each year between 2016 and 2040, average health of 60–65.5-year-olds of PHM 2016 and estimated health of 60–68 year-olds in 2040 of step 1 was interpolated linearly.

Scenario B, an improvement in health, is implemented as follows. We used data of respondents aged 55–75 of the PHM, and aged ≥16 years and over of the HIS, both categorized in similar five-year age categories. First, we interpolated the data of PHM assuming a linear age-related decrease in health between the ages of 55–60 and 65–70 years because we assume that there will be no (longer) adverse health effects of extended working lives. Prevalence of good health (men and women) and poor health (women) among 60–65-year-olds was calculated as the average of the 55–60- and 65–70-year-olds. Prevalence of poor health of 60–65- and 65–70-year-old men was calculated by applying the same percent change that was calculated in good health among men. The latter was necessary as the proportion of men in poor health was lower among the 65–70-year-olds compared to the 55–60-year-olds. Health, according to this reconstructed data, is denoted as PHM 2016*. Second, using HIS 1990–2017, the health status in the five-year age categories was modelled up to 2040 using logistic regression analysis in R. The age range of ≥16 of HIS was used to model more robust health trends for ages 60–65 and 65–70 in comparison to estimations where surrounding age categories were not included. Third, only the resulting fitted growth rates for age categories 60–65 and 65–70 from 2016 to 2040 were applied to the health status in the corresponding age categories of PHM 2016*, which resulted in the health estimates for 2040. Fourth, health status between 2016 and 2040 was interpolated linearly for both age categories. Fifth, the average health for age 60 up to the state pension age was calculated for each calendar year, taking into account the stepwise increase of the state pension age according to current law and expectations (Table 1).

Finally, both scenarios are expressed as the absolute number of persons over 60 years of age up until the state pension age that is expected in good, moderate and poor health in 2030 and 2040. This is done by multiplying the percentages by absolute numbers according to the population forecast 2018 of Statistics Netherlands [33].

### 3.2. Weighting

Both datasets are weighted to the non-institutionalized Dutch population of each corresponding calendar year. The weight factors are provided by Statistics Netherlands.

### 3.3. Historical Health Trend

The projection of health up to 2040 for Scenario B is based on analysis of the historical trend. We used HIS to determine this health trend in five-year age categories. We estimated two trends in health. Firstly, for the period 1997–2017, and secondly, for 1990–2017. The first was estimated because since 1997, the sample was randomly derived from non-institutionalized persons instead of non-institutionalized households, and thus, the trend may be discontinuous in 1997. This trend showed that health improved in the age categories 55–60, 65–70 and 70–75 years, but did not improve in the age category 60–65 years (Table 2). To have a longer historical series, the trend for 1990–2017 was also estimated. This trend showed that health improved in all age categories; the fitted growth rate necessary for Scenario B is derived from this trend.

## 4. Results

### 4.1. Scenarios

**Figure 1:**
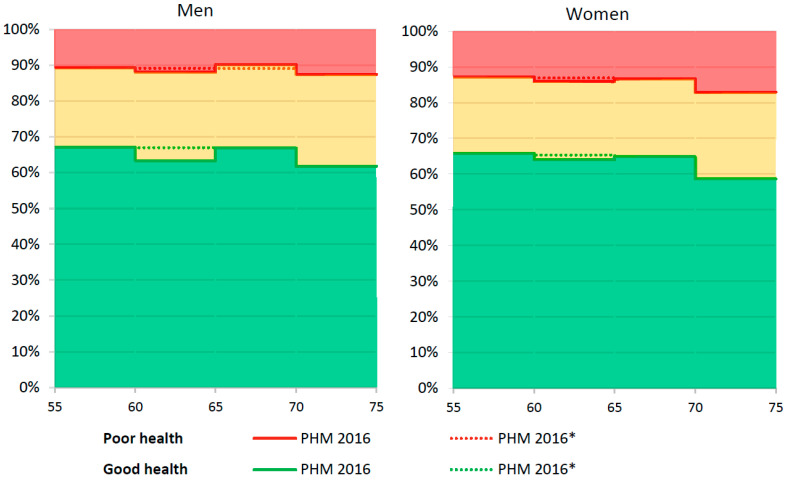
Health by age and gender in 2016, observed (denoted by PHM 2016) and reconstructed (denoted by PHM 2016*) Note. Prevalence of good health is below the green line and prevalence of poor health is above the red line, the intermediate area is prevalence of moderate health.

**Figure 2:**
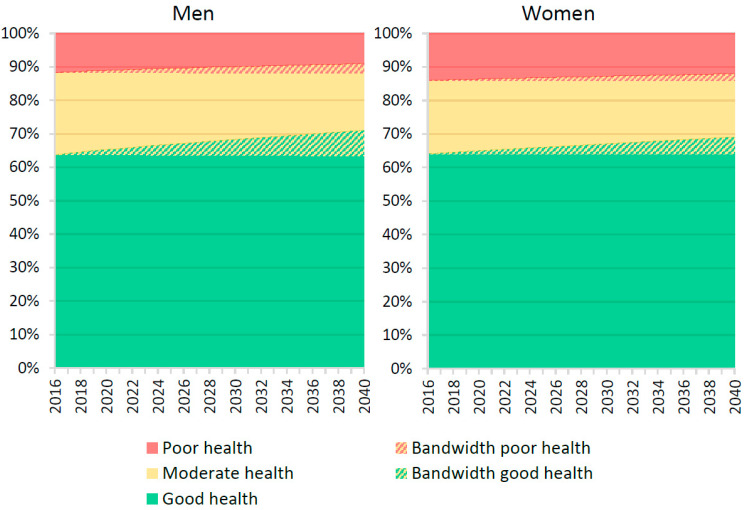
Projections in health for persons over 60 years of age up until the state pension age according to two scenarios (2016–2040).

Figure 1 presents health by five-year age categories for men and women, observed in the PHM 2016 (solid line) and reconstructed (dotted line; PHM 2016*). They serve as the starting point for scenarios A and B, respectively. Figure 2 presents the two scenarios up until 2040, which show the bandwidth for the percentage of people who are expected to be in good health (green-yellow striped area) and in poor health (red-yellow striped area).

#### 4.1.1. Good Health

Assuming a stable health trend (Scenario A), 63% of men aged 60–68 years are expected to be in good health in 2040 (Figure 2). Assuming an improving health trend (Scenario B), this is 71%. Among women aged 60–68 years, the expected proportion in good health ranges between 64% (Scenario A) and 69% (Scenario B).

#### 4.1.2. Poor Health

Assuming an improving health trend (Scenario B), 9% of 60–68-year-old men are expected to be in poor health in 2040 (Figure 2). Assuming a stable health trend (Scenario A) this is 12%. For 60–68-year-old women, the proportion in poor health is expected to range between 12% (Scenario B) and 14% (Scenario A).

#### 4.1.3. Population Estimates

In Table 3, the health distribution according to the two scenarios is applied to the Dutch population forecasts for 2030 and 2040. It shows that according to both scenarios, the absolute number of people aged 60 up to the state pension age in all three health categories will increase. The largest increases relative to 2016 are expected in 2030. Up to this year, the expected number of people aged 60–67.25 (state pension age in 2030) in good health will increase the most: with approximately 391,000 to 456,000. In addition, the number of 60–67.25-year-olds in moderate health (101,000–140,000) and poor health (52,000–77,000) will increase.

## 5. Discussion

The aim of this study is to explore the future health of Dutch adults from age 60 to the state pension age, up until 2040. Two possible scenarios are described and estimated: a stable health trend and an improving health trend. We assume that these two scenarios indicate a bandwidth between which health will develop.

### 5.1. Contextualizing the Results

According to the estimations, the majority of 60–68-year-olds is expected to be in good health (63–71% of men and 64–69% of women), but also a large proportion is expected to be in moderate health (17–28% of men and 17–24% of women) or poor health (9–12% of men and 12–14% of women) in 2040.

In view of the increased state pension, the results suggest that the majority of the older working-age population should be able to extend their working lives. People in moderate or poor health may have difficulty working longer due to their health problems. Both self-reported health and functional limitations have been shown to be associated with disability pension and unemployment, and self-reported health also with early retirement [7]. More recent research shows that poor health is becoming a weaker predictor of work exit, that the proportion of older workers with health problems increases and that older workers work more years with health problems [34,35]. It is not well understood how many people with health problems continue working because they are still capable or because they have no opportunity to stop working. Previous research shows that work ability and productivity are lower among older workers with health problems compared to those without health problems [32]. With the extension of working lives, this association may become even stronger.

Working beyond the age of 60 may also result in a greater proportion of people with health problems. This is suggested by previous research, but there is no conclusive evidence [12]. Our study provides indirect indications that health is negatively affected by the extension of working lives itself or by the way in which people were required to extend their working lives, i.e., by postponing the possibility for retirement several times. First, for the period 1997–2017, in which the employment rate increased by 31 percent points, the HIS shows a stable health trend for 60–65-year-olds, while the surrounding age categories show an improving health trend. Second, according to PHM 2016, 63% of men and 64% of women were in good health. The close similarity of these percentages is unexpected, because men generally report better health and functioning than women [36]. It may be due to the fact that in 2016, the employment rate among 60–65-year-old men was higher (63%) compared to women (43%) [18]. Third, PHM 2016 shows that in 2016, 60–65-year-olds were less healthy compared to 65–70-year-olds. This phenomenon has also been observed in 2013–2015 in several other European countries, i.e., in Belgium, Luxembourg, Denmark, Finland and Sweden [37]. It may mean either that health improves after retirement, which is supported by some studies but contradicted by others [38], or that health of the cohort of 65–70-year-olds was not as much affected by the extension of working lives five years earlier. After all, in 2011, employment rates of 60–65-year-olds were 14 percentage points lower compared to 2016, i.e., 39% and 53%, respectively [18].

### 5.2. Methodological Considerations

There is a great deal of uncertainty about how the health of over-60s will develop. One important uncertain factor is the unknown long-term health effect of the COVID-19 pandemic. On the one hand, the pandemic situation may have affected population health negatively. During the pandemic, people may have lost loved ones, experienced fear to get infected, experienced job insecurity or job loss, ran into financial problems, and experienced social isolation and loneliness. All these stressors have affected mental health negatively during the pandemic and might still afterwards [39,40,41]. Moreover, among some of the infected people, the infection resulted in long-lasting health complaints (i.e., post-COVID syndrome or post-intensive care syndrome) [42,43]. Lastly, the interruption of routine care and postponement of non-urgent elective treatments might contribute to increased future morbidity related to acute and chronic health conditions [44]. On the other hand, there have been some positive aspects of the pandemic situation. People experienced more tranquility in their daily lives as a result of a reduction of social activities and of noise and pollution in the physical environment [45,46]. In addition, work flexibility, with less commute involved and increased autonomy, was perceived as positive [46]. In the Netherlands, two thirds of the employees would like to keep working from home to a certain extent [47]. When people maintain this calmer lifestyle in the future, it can lower stress levels and may benefit health [46,48].

So far, there is no sufficient evidence for firm conclusions about long-term subjective health effects of the pandemic at the population level. Data from the HIS 2020 and 2021 show that both self-reported health and functional limitations slightly improved among 55–65- and 65–75-year-olds in comparison to 2016 [49,50]. As the scenarios in this study are solely based on actual observed historical health trends, we did not make estimations for a deteriorating health scenario.

#### Strengths and Limitations

The first scenario is based on the historical stable health trends found by two studies using different Dutch samples in this age category [13,16]. This may be limited evidence, and only covering functional limitations and not self-reported health, but the stable trend is also supported by the results of the current study of the health trend of 60–65-year-olds in HIS from 1997 to 2017.

The second scenario is based on the improving health trend that is expected for 60–65-year-olds, if this age category follows the same trend as the surrounding ages according to the Dutch Public Health Foresight Study [6]. To estimate the extent of improvement, we used the health trend in five-year age categories from HIS 1990–2017 to model health up to 2040, and applied the modelled trend of 60–65- and 65–70-year-olds to the health status of 60–68-year-olds of the PHM 2016. Although using historical health trends to make projections for future health is the current state of the art [51], it has the disadvantage that it does not take into account developments in educational level, employment status, lifestyle or health care.

The data used have some advantages and disadvantages. The strength of the PHM is its large sample size, which provides estimations of health in these specific age categories with more accuracy compared to HIS. The strength of the HIS is the availability of historical data, which allows us to estimate longitudinal health trends. It is a large survey, allowing trend estimations also in narrow age categories around the state pension age. However, the sample is too small to accurately estimate health in specific age groups in each individual year. Moreover, stratification by other relevant factors such as educational level is not possible when using five-year age categories. It should be noted that low educated people are generally in poorer health compared to highly educated people [52,53]. In addition, low educated workers work more often in physically demanding jobs [52] and have few resources to exit the workforce early [15]. The extension of working lives may, therefore, be a greater challenge for low educated people, and socio-economic differences threaten to widen.

By combining the historical health trends from the HIS with the point prevalence of health from the PHM, we used the best available data from the Netherlands. However, combining the two datasets for the estimates in scenario B may raise the question of its justification. In both datasets, the exact same health variables were used and both datasets included randomly selected representative samples from the Dutch non-institutionalized population. One notable difference is that age at interview date was used for HIS-respondents and age at the 31st of December was used for PHM-respondents. This difference may have had an effect on the results, although we assume that it would not have changed the results greatly if the exact date had been available, because prevalence of good, moderate and poor health fluctuated somewhat between successive age years. By using five-year age categories, we attempted to level out this fluctuation.

Health was measured by a combination of two often-used indicators: self-reported health and limitations in mobility, hearing or seeing [30,31]. This combination has not been used before and its limitation is that the results are not comparable with results from other studies. However, this combined health indicator is more suitable than each indicator separately because it gives a more comprehensive indication of health status. Self-reported health may also include a mental health component, while self-reported limitations may be more indicative of the physical capability of continuing work [54]. Moreover, a preliminary analysis showed that the combined health indicator was indeed more predictive of labor market participation (expressed in currently working, non-working, receiving disability pension and retired) compared to each single indicator.

### 5.3. Implications for Practice and Further Research

In this study, we explored future health of over-60s in The Netherlands. Similar developments in health may be expected in other countries, as the extension of working lives is taking place in many countries, and several countries are noticing a stagnating health trend among 60–65-year-olds [8,9,10,11,12,13]. Therefore, the implications for practice and research may also apply to these countries.

What do the explorations for future health of Dutch adults from age 60 to the state pension age imply for the current policy on the state pension age? The scenarios show that the majority of the population in the older working-ages will be in good health. The proportions of over-60-year-olds in moderate and poor health may remain stable or decrease in the coming years. However, when the proportions are expressed in absolute numbers it turns out that the total number of over-60-year-olds in moderate and poor health will increase according to both scenarios. In 2016, there were 417,000 over-60s in poor and moderate health before state pension age. By 2030, this number is expected to increase to at least 570,000. This is a result of large birth cohorts from the 1960s reaching ages 60+ of the raised state pension age. In 2040, that number will drop to at least 475,000.

People in both moderate and poor health have an increased risk to drop out early from the labor force, or to continue working reluctantly while their work ability and productivity decreases [7,32]. These situations are undesirable from both a societal and an individual point of view. It is, therefore, necessary to continue taking measures to keep workers healthy and employable up until the state pension age. Research on effective interventions in this regard has increased in the past twenty years, but results are inconclusive and effect sizes small [55,56,57]. More research is needed on effective interventions, with a special focus on more vulnerable categories such as lower educated workers and the growing category of self-employed workers [56].

It is also important to monitor the developments in health of over-60-year-olds during the coming years. The state pension age in the Netherlands had been raised by only six months in 2016 and short- and long-term health effects of working beyond age 65 are not known yet. Potentially, working at these higher ages is harmful, especially in occupations that are physically and psychosocially demanding [19,20]. Moreover, if the stable trend among 60–68-year-olds continues in the coming years, it is unlikely that the health in age categories ≥70 years will continue to improve. A stabilization or decline in the health of over-70s should therefore, be anticipated. This could have far-reaching consequences, for example for (informal) care, health care costs, life expectancy and the quality of life of individuals.

## 6. Conclusions

This study’s explorations suggest that the majority of the future older working-age population (aged 60–68 years) will be in good health and, thus, should be able to extend their working lives, but also that a substantial share of people will be in moderate or poor health and, thus, may have difficulty working longer. Policy aiming at sustainable employability will, therefore, remain important, even in the case of the most favorable health trends. Moreover, health of future adults aged 60+ should be monitored as potential adverse health effects of the extension of working lives are not clear yet.

## Figures and Tables

**Table 1:** State pension age.

Year	State Pension Age *
2016	65 + 6 months
2017	65 + 9 months
2018	66
2019–2021	66 + 4 months
2022	66 + 7 months
2023	66 + 10 months
2024–2027	67
2028–2030	67 + 3 months
2031–2033	67 + 6 months
2034–2037	67 + 9 months
2038–2040	68

* Legally defined until 2027; state pension ages in later years are current expectations dependent on the life expectancy at age 65.

**Table 2:** Fitted growth rate for separate age categories for periods 1990–2017 and 1997–2017 (based on HIS).

	1990–2017		1997–2017	
	Men	Women	Men	Women
55–60				
Good health	0.144 *	0.073 *	0.055 *	0.084 *
Moderate Health	−0.110 *	−0.047 *	−0.053 *	−0.046
Poor health	−0.034 *	−0.026	−0.002	−0.038
60–65				
Good health	0.047 *	0.077 *	0.001	0.017
Moderate Health	−0.013	−0.071 *	−0.003	−0.026
Poor health	−0.034 *	−0.006	0.002	0.009
65–70				
Good health	0.122 *	0.158 *	0.088 *	0.114 *
Moderate Health	−0.083 *	−0.065*	−0.060 *	−0.074 *
Poor health	−0.040 *	−0.095*	−0.028	−0.040
70–75				
Good health	0.111 *	0.169 *	0.082 *	0.171 *
Moderate Health	−0.062 *	−0.057 *	−0.055	−0.084 *
Poor health	−0.049 *	−0.112 *	−0.027	−0.089 *

* Fitted growth rate increases/decreases significantly at the 5% level.

**Table 3:** Health distribution among men and women from age 60 up until the state pension age, in 2016, 2030 and 2040 (absolute numbers ×1000 and growth rates).

	2016	2030		2040	
		Scenario A	Scenario B	Scenario A	Scenario B
Men					
Good health	367	+193 (+53%)	+232 (+63%)	+124 (+34%)	+183 (+50%)
Moderate health	142	+73 (+52%)	+49 (+35%)	+50 (+35%)	+13 (+9%)
Poor health	67	+34 (+50%)	+20 (+29%)	+25 (+37%)	+2 (+4%)
Total	576	+300 (+52%)	+300 (+52%)	+199 (+34%)	+199 (+34%)
Women					
Good health	371	+198 (+53%)	+224 (+60%)	+147 (+40%)	+188 (+51%)
Moderate health	127	+67 (+53%)	+52 (+41%)	+51 (+40%)	+26 (+21%)
Poor health	81	+43 (+53%)	+32 (+40%)	+33 (+40%)	+16 (+20%)
Total	579	+308 (+53%)	+308 (+53%)	+230 (+40%)	+230 (+40%)
Men and women					
Good health	738	+391 (+53%)	+456 (+62%)	+271 (+37%)	+372 (+50%)
Moderate health	268	+140 (+52%)	+101 (+38%)	+100 (+37%)	+39 (+14%)
Poor health	149	+77 (+52%)	+52 (+35%)	+58 (+39%)	+19 (+12%)
Total	1154	+608 (+53%)	+608 (+53%)	+429 (+37%)	+429 (+37%)

## Data Availability

Restrictions apply to the availability of these data. Data was obtained from Statistics Netherlands and are available on request at https://www.cbs.nl/en-gb/onze-diensten/customised-services-microdata/microdata-conducting-your-own-research. Additionally, permission is required for use of the Public Health Monitor 2016, which can be requested at https://www.monitorgezondheid.nl/data-aanvraag.
